# Anti-inflammatory, tissue remodeling, immunomodulatory, and anticancer activities of oregano (*Origanum vulgare*) essential oil in a human skin disease model

**DOI:** 10.1016/j.biopen.2017.02.005

**Published:** 2017-03-03

**Authors:** Xuesheng Han, Tory L. Parker

**Affiliations:** dōTERRA International, LLC, 389 S. 1300 W., Pleasant Grove, UT 84062, USA

**Keywords:** Carvacrol, Antiproliferation, Skin health, Vascular cell adhesion molecule-1, Monokine induced by gamma interferon, Anticancer

## Abstract

The use of oregano (*Origanum vulgare*) essential oil (OEO) has become popular in skin care products. However, scientific research regarding its effects on human skin cells is scarce. In this study, we investigated the biological activity of a commercially available OEO, which is high in carvacrol content, in a human skin cell disease model. OEO induced marked antiproliferative effects and significantly inhibited several inflammatory biomarkers, including monocyte chemoattractant protein 1 (MCP-1), vascular cell adhesion molecule 1 (VCAM-1), intracellular cell adhesion molecule 1 (ICAM-1), interferon gamma-induced protein 10 (IP-10), interferon-inducible T-cell alpha chemoattractant (I-TAC), and monokine induced by gamma interferon (MIG). OEO also significantly inhibited tissue remodeling biomarkers, namely collagen I, collagen III, epidermal growth factor receptor (EGFR), matrix metalloproteinase 1 (MMP-1), plasminogen activator inhibitor 1 (PAI-1), tissue inhibitor of metalloproteinase (TIMP) 1 and 2. An immunomodulatory biomarker, macrophage colony-stimulating factor (M-CSF), was also strongly inhibited by OEO treatment. In addition, OEO significantly modulated global gene expression and altered signaling pathways, many of which are critical in inflammation, tissue remodeling, and cancer signaling processes. These findings along with existing studies largely support the anti-inflammatory, tissue remodeling, immunomodulatory, and anticancer activities of OEO. In conclusion, this study provides the first evidence of the biological activity of OEO in human dermal fibroblasts. We suggest that OEO, with carvacrol as the major active component, is a promising candidate for use in skin care products with anti-inflammatory and anticancer properties.

## Highlights

• Oregano essential oil (OEO) was anti-proliferative in human dermal fibroblasts.
• OEO inhibited inflammatory biomarkers MCP-1, VCAM-1, ICAM-1, IP-10, I-TAC, and MIG.
• OEO inhibited M-CSF, collagen I, collagen III, EGFR, MMP-1, PAI-1, TIMP-1 and -2.
• OEO impacted critical genes for inflammation, wound healing, and cancer signaling.

## 1 Introduction

Oregano (*Origanum vulgare*) essential oil (OEO^1^) has a high content of carvacrol, a monoterpenoid phenol. OEO and carvacrol have been studied for a variety of biological and pharmacological properties, including antioxidant, antibacterial, antifungal, anticancer, and anti-inflammatory properties [1]. However, studies on the effects of OEO in human skin cells are scarce, even though OEO has gained popularity in skin care products. To the best of our knowledge, the biological activity of OEO in human dermal fibroblast cells has not been evaluated.

The aim of this study was to evaluate the biological activity of a commercially available OEO with a high carvacrol content in a validated human dermal fibroblast cell line, which was designed to model the disease pathology of chronic inflammation and fibrosis. We first analyzed the impact of OEO on 17 important protein biomarkers closely related to inflammation and tissue remodeling processes. We also studied its effect on regulating genome-wide gene expression. The study provides important evidence supporting the anti-inflammatory, tissue remodeling, and anticancer activities of OEO.

## 2 Materials and methods

All experiments were conducted in a BioMAP HDF3CGF system, a cell culture of human dermal fibroblasts that is designed to model chronic inflammation and fibrosis in a robust and reproducible way. The system consists of three components: a cell type, stimuli to create the disease environment, and a set of biomarker (protein) readouts to examine how treatments affect that disease environment [2], [3].

### 2.1 Cell culture

Primary human neonatal fibroblasts were obtained as described previously [4]. These cells were plated in a 96-well format and cultured under low-serum conditions for 24 h. Then they were stimulated with a mixture of interleukin-1β, tumor necrosis factor-α, interferon-ϒ, basic fibroblast growth factor, epidermal growth factor, and platelet-derived growth factor. Cell culture and stimulation conditions for the HDF3CGF assays were performed as described in detail elsewhere [4], [5].

### 2.2 Protein-based readouts

A direct enzyme-linked immunosorbent assay (ELISA) was used to measure the biomarker levels of cell-associated and cell membrane targets. Soluble factors from supernatants were quantified using homogeneous time-resolved fluorescence detection, bead-based multiplex immunoassay, or capture ELISA. Overt adverse effects of the test agents on cell proliferation and viability (i.e., cytotoxicity) were measured using a sulforhodamine B assay. For proliferation assays, cells were cultured and then assayed after 72 h, which was optimized for the HDF3CGF system. Detailed information has been described elsewhere [4]. Measurements were performed in triplicate wells, and a glossary of the biomarkers used in this study is provided in Supplementary Table S1.

Quantitative biomarker data are presented as the mean log_10_ relative expression level (compared to the respective mean vehicle control value) ± standard deviation of triplicate measurements. Differences in biomarker levels between OEO- and vehicle-treated cultures were tested for significance with the unpaired Student's t test. A p value < 0.05, with an effect size of at least 10% (more than 0.05 log_10_ ratio units), was regarded as statistically significant.

### 2.3 RNA isolation

Total RNA was isolated from cell lysates using the Zymo *Quick-RNA* MiniPrep kit (Zymo Research Corporation, Irvine, CA), according to the manufacturer's instructions. The RNA concentration was determined using a NanoDrop ND-2000 (Thermo Fisher Scientific, Waltham, MA, USA) instrument. RNA quality was assessed with a Bioanalyzer 2100 instrument (Agilent Technologies, Santa Clara, CA, USA) and an Agilent RNA 6000 Nano Kit. All samples had an A260/A280 ratio between 1.9 and 2.1, and an RNA Integrity Number score >8.0.

### 2.4 Microarray analysis for genome-wide gene expression

A 0.0037% (v/v) concentration of OEO was tested for its effect on the expression of 21,224 genes in the HDF3CGF system after a 24-h treatment. Samples for microarray analysis were processed by Asuragen, Inc. (Austin, TX, USA), according to the company's standard operating procedures. Biotin-labeled cRNA was prepared from 200 ng of total RNA with an Illumina TotalPrep RNA Amplification kit (Thermo Fisher Scientific) and one round of amplification. The cRNA yields were quantified via UV spectroscopy, and the distribution of transcript sizes was assessed using the Agilent Bioanalyzer 2100. Labeled cRNA (750 ng) was used to probe Illumina Human HT-12 v4 Expression BeadChips (Illumina, Inc., San Diego, CA, USA). Hybridizing, washing, staining with streptavidin-conjugated cyanine-3, and scanning of the Illumina arrays were performed according to the manufacturer's instructions. Illumina BeadScan software was used to produce the data files for each array; raw data were extracted using Illumina BeadStudio software.

Raw data were uploaded into R [5] and analyzed for quality-control metrics using the *beadarray* package [6]. Data were normalized using quantile normalization [7], then re-annotated and filtered to remove probes that were nonspecific or mapped to intronic or intragenic regions [8]. The remaining probe sets comprised the data set for the remainder of the analysis. Fold-change expression for each value was calculated as the log_2_ ratio of OEO to the vehicle control. These fold-change values were uploaded to Ingenuity Pathway Analysis (IPA, QIAGEN, Redwood City, CA, USA, www.qiagen.com/ingenuity) to generate the network and pathway analyses.

### 2.5 Reagents

OEO (dōTERRA, Pleasant Grove, UT, USA) was diluted in dimethyl sulfoxide (DMSO) to 8× the specified concentrations (the final DMSO concentration in the culture media was no more than 0.1% [v/v]); 25 μL of each 8× solution was added to the cell culture to a final volume of 200 μL. DMSO (0.1% [v/v]) served as the vehicle control. The gas chromatography-mass spectrometry analysis of OEO indicated that its major chemical constitute (i.e., >5%) was carvacrol (78%).

## 3 Results and discussion

### 3.1 Bioactivity profile of OEO in pre-inflamed human dermal fibroblasts

**Fig. 1:**
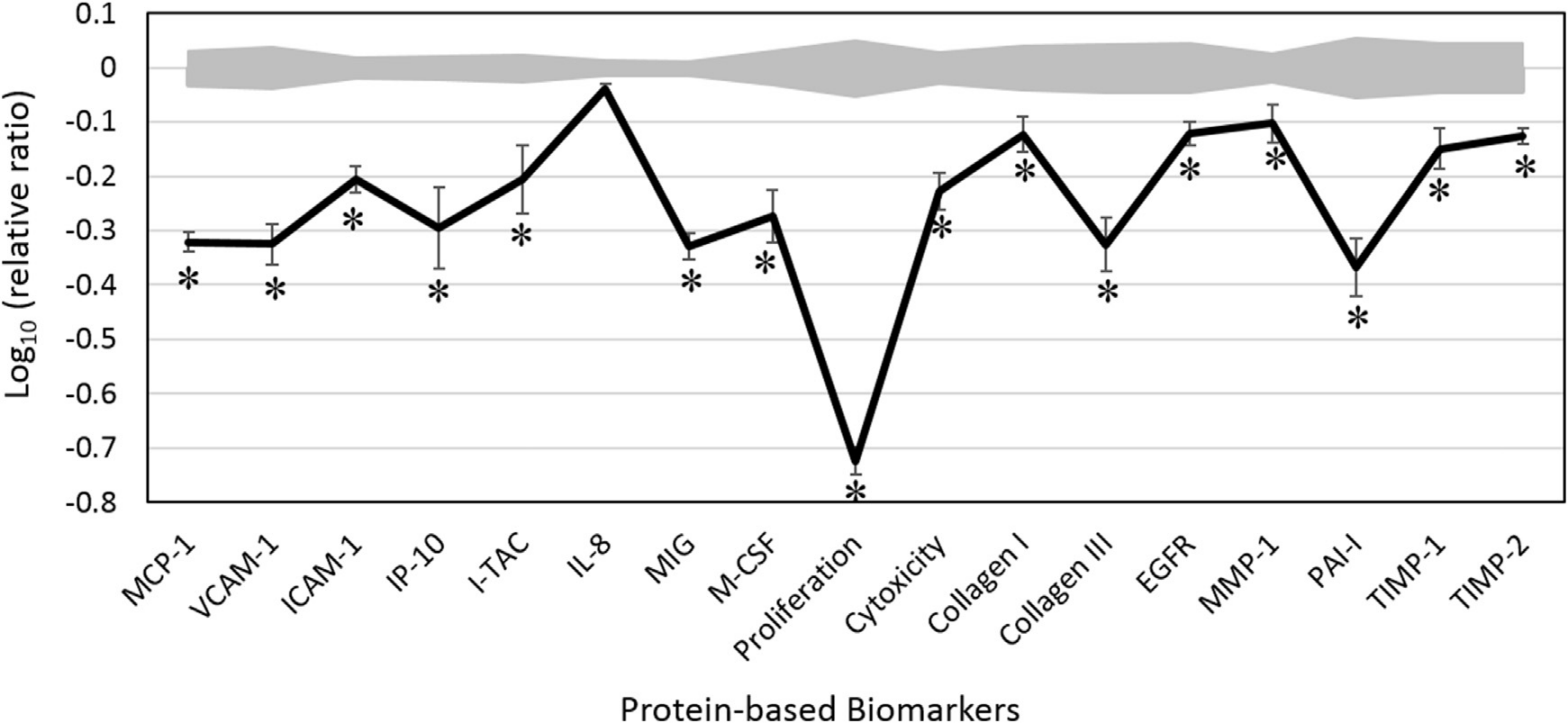
The bioactivity profile of oregano essential oil (OEO; 0.0037%, v/v in dimethyl sulfoxide, DMSO) using the BioMAP System HDF3CGF. The x-axis denotes protein-based biomarker readouts. The y-axis denotes the relative expression levels of biomarkers compared to vehicle control values, in log form. Vehicle control values are shaded in gray, denoting the 95% confidence level. The asterisks (*) indicate the biomarkers designated with “key activity,” i.e., the biomarker values were significantly different (p < 0.05) after cell treatment with 0.0037% OEO, compared to those of vehicle controls, with an effect size of at least 10% (more than 0.05 log ratio units). MCP-1, monocyte chemoattractant protein; VCAM-1, vascular cell adhesion molecule 1; ICAM-1, intracellular cell adhesion molecule 1; IP-10, interferon gamma-induced protein 10; I-TAC, interferon-inducible T-cell alpha chemoattractant; IL-8, interleukin-8; MIG, monokine induced by gamma interferon; EGFR, epidermal growth factor receptor; M-CSF, macrophage colony-stimulating factor; MMP-1, matrix metalloproteinase 1; PAI-1, plasminogen activator inhibitor 1; TIMP, tissue inhibitor of metalloproteinase.

We analyzed the activity of OEO in a dermal fibroblast system, which features the disease microenvironment of inflamed human skin cells with boosted inflammation and immune responses. Four concentrations of OEO (0.011, 0.0037, 0.0012, and 0.00041%, v/v) were tested for cell viability. The highest concentration (0.011%) was overtly cytotoxic; thus, only the three lower concentrations were included for further analysis. Biomarkers were designated as having key activity if their values were significantly different (p < 0.05) after cell treatment with 0.0037% OEO, compared to those of vehicle controls, with an effect size of at least 10% (more than 0.05 log ratio units) (Fig. 1).

OEO treatment inhibited all 17 of the biomarkers studied. It showed significant antiproliferative activity to dermal fibroblast cells. OEO significantly decreased the levels of several inflammatory biomarkers, including monocyte chemoattractant protein 1 (MCP-1), vascular cell adhesion molecule 1 (VCAM-1), intracellular cell adhesion molecule 1 (ICAM-1), interferon gamma-induced protein 10 (IP-10), interferon-inducible T-cell alpha chemoattractant (I-TAC), and monokine induced by gamma interferon (MIG). All studied tissue remodeling biomarkers, namely collagen I, collagen III, macrophage colony-stimulating factor (M-CSF), epidermal growth factor receptor (EGRF), matrix metalloproteinase 1 (MMP-1), plasminogen activator inhibitor 1 (PAI-1), tissue inhibitor of metalloproteinase (TIMP) 1, and TIMP2 were also significantly decreased by OEO treatment. OEO only slightly inhibited interleukin 8, a pro-inflammatory chemokine. Of note, the inhibitory effect of OEO on these biomarkers in the highly inflamed skin cells was concentration dependent.

The significant inhibitory effect of OEO on proliferation as well as these inflammatory and tissue remodeling biomarkers indicates that OEO may possess anti-inflammatory, immunomodulatory, tissue remodeling, and pro-wound healing properties. These findings are largely supported by published research regarding OEO and its major active component, carvacrol.

Carvacrol has been shown to suppress proliferation and induce apoptosis in porcine enterocytes and lymphocytes in a significant manner [9]. Recently, Marrelli et al. have reported that OEO with a high carvacrol content has antiproliferative effects on human colon and liver cancer cells [10], suggesting its potential to combat cancer.

Carvacrol has been found to inhibit inflammation induced by carrageenan and tumor necrosis factor-alpha in a mouse model [11]. In addition, Silva and colleagues have reported the anti-inflammatory and anti-ulcer activities of carvacrol in an experimental model of edema; their results suggest that carvacrol probably interferes with inflammatory mediators and thus promotes the healing process for ulcers [12]. Scientists in Russia have found that OEO increases the content of antibody-forming lymphocytes in the mouse spleen in response to ionizing radiation, suggesting the anti-inflammatory and immune-enhancing properties of OEO [13]. Most recently, Zou and colleagues have evaluated the mechanism of how OEO promotes the intestinal barrier integrity in a pig model and found that OEO significantly impacts mitogen-activated protein kinase, protein kinase B, and nuclear factor κB signaling pathways and inhibits the expression of inflammatory cytokines, supporting the anti-inflammatory and immunomodulatory properties of OEO [14].

### 3.2 Effects of OEO on genome-wide gene expression

Next, we analyzed the effect of 0.0037% OEO (the highest tested concentration that was noncytotoxic to these cells) on the RNA expression of 21,224 genes in the HDF3CGF system. The results showed a robust and diverse effect of OEO on regulating human genes, with many downregulated genes and many upregulated genes. Among the 200 most-regulated genes (with a fold-change ratio of expression over vehicle control ≥ |1.5|) by OEO, most of them (115 out of 200 genes) were significantly downregulated, and the remaining were upregulated (Table S2). A cross-comparison indicated that MIG was significantly inhibited by OEO at both the protein and gene levels, suggesting that MIG might be a key mediator for the activity of OEO in the human skin disease model.

**Fig. 2:**
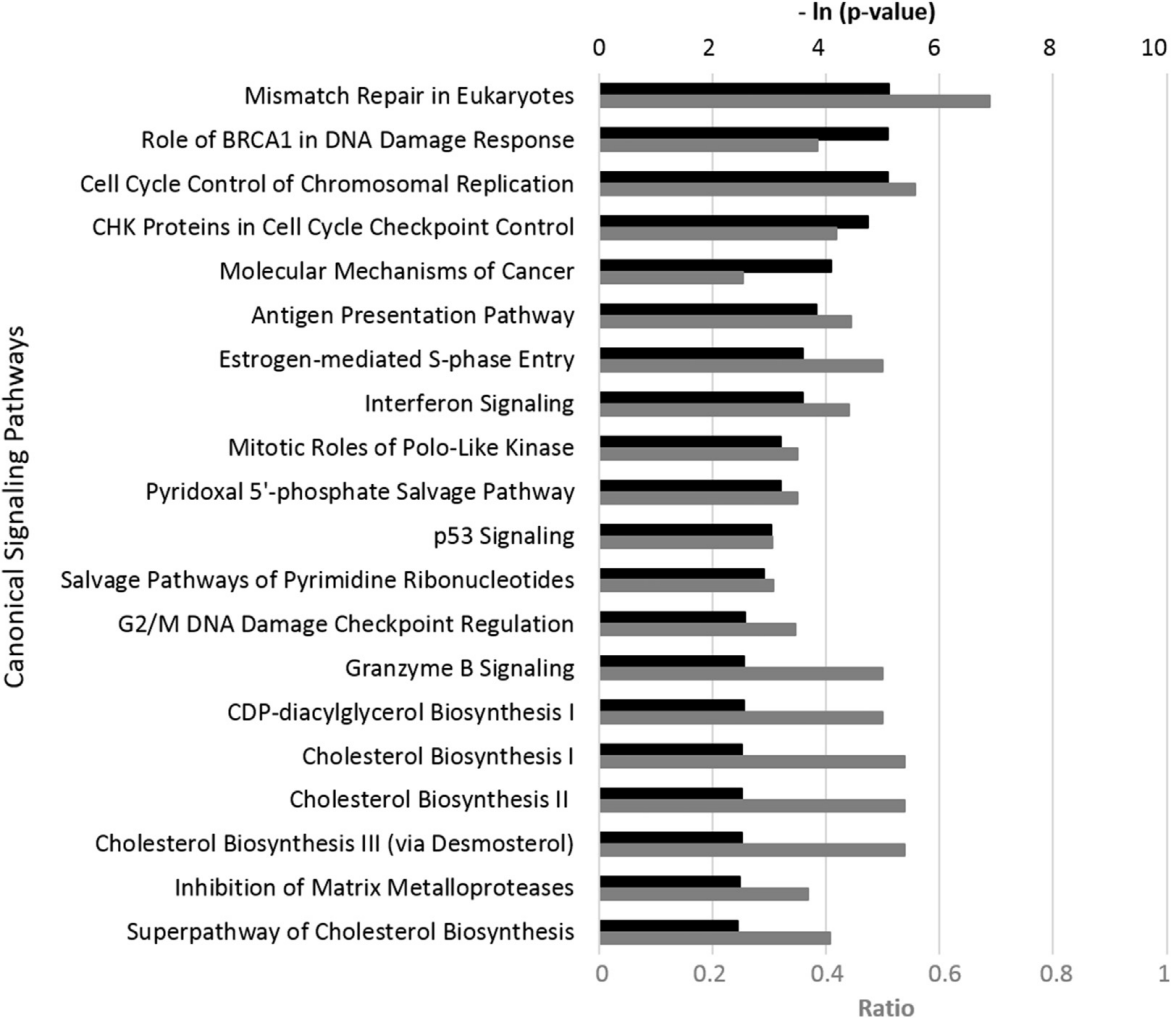
The top 20 canonical pathways matching the gene expression bioactivity profile of oregano essential oil (OEO) in the HDF3CGF system, produced via Ingenuity Pathway Analysis (IPA, www.qiagen.com/ingenuity). Each p-value was calculated with the right-tailed Fisher's Exact Test. The p-value measures the likelihood that the observed association between a specific pathway and the dataset is due to random chance. The smaller the p value (the bigger – ln (p-value), indicated by the black bars) that the pathway has, the more significantly it matches with the bioactivity of OEO. The ratio, indicated by each gray bar, was calculated by taking the number of genes from the OEO dataset that participate in a canonical pathway and dividing it by the total number of genes in that pathway. BRCA1, breast cancer type 1; CHK, checkpoint kinase; p53, tumor protein p53; CDP, cytidine diphosphate.

IPA analysis showed that the bioactivity of OEO was significantly matched with many canonical signaling pathways from the literature-validated database (Fig. 2). Many of these pathways play critical roles in inflammation, cell cycle control, DNA damage response, and cancer development and progression. For instance, the top four matched pathways are all closely related to cell cycle control and DNA damage response (Tables S3–6). The overall inhibitory effect of OEO on these signaling pathways suggests that OEO may play a role in regulating the cell cycle and molecular mechanisms of cancer.

Bostancıoğlu and colleagues have assessed the antiangiogenic and anticancer potential of OEO with a high-carvacrol content in rat adipose tissue endothelial cells (RATECs) and c-H-ras-transformed rat embryonic fibroblasts (5RP7 cells); their results showed that OEO markedly inhibits the cell viability and induces apoptosis of 5RP7 cells as well as blocks *in vitro* tube formation and migration of RATECs, suggesting that OEO has potential as an anticancer agent [15]. Similarly, Liang and Lu have reported that carvacrol induces apoptosis in human glioblastoma cells [16]. More interestingly, Guimarães and colleagues have recently found that encapsulation of carvacrol with β-cyclodextrin significantly reduces hyperalgesia in mice with tumors, suggesting that OEO may be a useful option for pain management [17].

Collectively, the current study along with the existing literature suggest that OEO (with carvacrol as the major active component) possesses promising anti-inflammatory, tissue remodeling, immunomodulatory, and anticancer properties. The data obtained from the current *in vitro* study cannot be directly applied to more complex human systems. Further research into the biological and pharmacological mechanisms of action of OEO is recommended.

## 4 Conclusions

To the best of our knowledge, this study is the first to evaluate the biological activity of OEO in pre-inflamed human dermal fibroblasts. OEO showed significant antiproliferative activity. It inhibited the levels of many inflammatory and tissue remodeling biomarkers, including MCP-1, VCAM-1, ICAM-1, IP-10, I-TAC, IP-10, MIG, collagen I, collagen III, M-CSF, EGRF, MMP-1, PAI-1, TIMP1, and TIMP2. In addition, genome-wide gene expression analysis demonstrated that OEO exerted a robust and diverse impact on many genes and signaling pathways, many of which are critically involved in inflammation, tissue remodeling, and cancer signaling processes. These results are largely consistent with studies reporting on the anti-inflammatory, wound healing, and anticancer potential of OEO. Therefore, OEO, with carvacrol as the major active component, is a promising candidate for use in skin care products with anti-inflammatory and anticancer properties.

## Conflicts of interest

X.H. and T.P. are employees of dōTERRA, where the study agent OEO was manufactured.

## Footnotes

1 OEO, oregano essential oil.
