# Supplementary material for: Anti-inflammatory, tissue remodeling, immunomodulatory, and anticancer activities of oregano (Origanum vulgare) essential oil in a human skin disease model
Source: Biochim Open. 2017 Mar 3;4:73–7. doi: 10.1016/j.biopen.2017.02.005 (PMC5801825; doi:10.1016/j.biopen.2017.02.005)

Table S1. Glossary of biomarkers of system HDF3CGF used in the study

| **Readout** | **Description** |
| --- | --- |
| **CCL2/MCP-1** | MCP-1 system is a chemokine that mediates recruitment of monocytes and T cells into sites of inflammation. MCP-1 is categorized as an inflammation-related activity in the HDF3CGF system modeling Th1 inflammation involved in wound healing and matrix remodeling. |
| **CD106/VCAM-1** | VCAM-1 is a cell adhesion molecule that mediates adhesion of monocytes and T cells to endothelial cells. VCAM-1 is categorized as an inflammation-related activity. |
| **CD54/ICAM-1** | ICAM-1 is a cell adhesion molecule that mediates leukocyte-endothelial cell adhesion and leukocyte recruitment. ICAM-1 is categorized as an inflammation-related activity. |
| **Collagen I** | Collagen I is involved in tissue remodeling and fibrosis, and is the most common fibrillar collagen that is found in skin, bone, tendons and other connective tissues. Collagen I is categorized as a tissue remodeling-related activity. |
| **Collagen III** | Collagen III is an extracellular matrix protein and fibrillar collagen found in extensible connective tissues (skin, lung and vascular system) and is involved in cell adhesion, cell migration, tissue remodeling. Collagen III is categorized as a tissue remodeling-related activity. |
| **CXCL10/IP-10** | IP-10 is a chemokine that mediates T cell, monocyte and dendritic cell chemotaxis. IP-10 is categorized as an inflammation-related activity. |
| **CXCL11/I-TAC** | I-TAC is a chemokine that mediates T cell and monocyte chemotaxis. I-TAC is categorized as an inflammation-related activity. |
| **CXCL8/IL-8** | IL-8 is a chemokine that mediates neutrophil recruitment into acute inflammatory sites. IL-8 is categorized as an inflammation-related activity. |
| **CXCL9/MIG** | MIG is a chemokine that mediates T cell recruitment. MIG is categorized as an inflammation-related activity. |
| **EGFR** | EGFR is a cell surface receptor for epidermal growth factor involved in cell proliferation during development as well as tumor growth. EGFR is involved in Epithelial cell proliferation, epithelial cell differentiation keratinocyte proliferation, tissue remodeling. EGFR is categorized as a tissue remodeling-related activity. |
| **M-CSF** | M-CSF is a secreted and cell surface cytokine that mediates macrophage differentiation. M-CSF is categorized as an immune modulation-related activity. |
| **MMP-1** | MMP-1 is an interstitial collagenase that degrades collagens I, II and III and is involved in the process of tissue remodeling. MMP-1 is categorized as a tissue remodeling-related activity. |
| **PAI-I** | PAI-I is a serine proteinase inhibitor and inhibitor of tissue plasminogen activator (tPA) and urokinase (uPA) and is involved in tissue remodeling and fibrinolysis. PAI-I is categorized as a tissue remodeling-related activity. |
| **Proliferation_72hr** | Proliferation_72hr in the HDF3CGF system is a measure of dermal fibroblast proliferation which is important to the process of wound healing and fibrosis. |
| **SRB** | SRB is a measure of the total protein content of dermal fibroblasts. Cell viability of adherent cells is measured by Sulforhodamine B (SRB) staining, a method that determines cell density by measuring total protein content of test wells. |
| **TIMP-1** | TIMP-1 is a tissue inhibitor of matrix metalloprotease-7 (MMP-7) and other MMPs, and is involved in tissue remodeling, angiogenesis and fibrosis. TIMP-1 is categorized as a tissue remodeling-related activity. |
| **TIMP-2** | TIMP-2 is a tissue inhibitor of matrix metalloproteases and is involved in tissue remodeling, angiogenesis and fibrosis. TIMP-2 is categorized as a tissue remodeling-related activity. |

Table S2. Top 200 genes impacted by OEO (fold change in log_2_ ratio form)

| **Illumina Gene ID** | **Fold Change** | **Definition** |
| --- | --- | --- |
| RSAD2 | 10.61 | Homo sapiens radical S-adenosyl methionine domain containing 2 (RSAD2), mRNA. |
| IFI27 | 6.19 | Homo sapiens interferon, alpha-inducible protein 27 (IFI27), transcript variant 2, mRNA. |
| IFIT1 | 6.07 | Homo sapiens interferon-induced protein with tetratricopeptide repeats 1 (IFIT1), transcript variant 2, mRNA. |
| IFIT3 | 4.87 | Homo sapiens interferon-induced protein with tetratricopeptide repeats 3 (IFIT3), mRNA. |
| OASL | 4.85 | Homo sapiens 2'-5'-oligoadenylate synthetase-like (OASL), transcript variant 2, mRNA. |
| MX2 | 4.70 | Homo sapiens myxovirus (influenza virus) resistance 2 (mouse) (MX2), mRNA. |
| HES4 | 4.50 | Homo sapiens hairy and enhancer of split 4 (Drosophila) (HES4), mRNA. |
| LOC387763 | 3.64 | PREDICTED: Homo sapiens hypothetical LOC387763 (LOC387763), mRNA. |
| USP18 | 3.50 | Homo sapiens ubiquitin specific peptidase 18 (USP18), mRNA. |
| TNFSF10 | 3.48 | Homo sapiens tumor necrosis factor (ligand) superfamily, member 10 (TNFSF10), mRNA. |
| OAS1 | 3.46 | Homo sapiens 2',5'-oligoadenylate synthetase 1, 40/46kDa (OAS1), transcript variant 3, mRNA. |
| OAS1 | 3.46 | Homo sapiens 2',5'-oligoadenylate synthetase 1, 40/46kDa (OAS1), transcript variant 3, mRNA. |
| ISG15 | 3.43 | Homo sapiens ISG15 ubiquitin-like modifier (ISG15), mRNA. |
| OASL | 3.39 | Homo sapiens 2'-5'-oligoadenylate synthetase-like (OASL), transcript variant 1, mRNA. |
| LOC100129681 | 3.29 | PREDICTED: Homo sapiens similar to NPC-A-7 (LOC100129681), mRNA. |
| OAS1 | 3.28 | Homo sapiens 2',5'-oligoadenylate synthetase 1, 40/46kDa (OAS1), transcript variant 2, mRNA. |
| CMPK2 | 3.20 | Homo sapiens cytidine monophosphate (UMP-CMP) kinase 2, mitochondrial (CMPK2), nuclear gene encoding mitochondrial protein, mRNA. |
| BST2 | 3.19 | Homo sapiens bone marrow stromal cell antigen 2 (BST2), mRNA. |
| MAP2 | 3.06 | Homo sapiens microtubule-associated protein 2 (MAP2), transcript variant 1, mRNA. |
| MAP2 | 2.90 | Homo sapiens microtubule-associated protein 2 (MAP2), transcript variant 2, mRNA. |
| TNFSF13B | 2.87 | Homo sapiens tumor necrosis factor (ligand) superfamily, member 13b (TNFSF13B), transcript variant 1, mRNA. |
| IFIT2 | 2.86 | Homo sapiens interferon-induced protein with tetratricopeptide repeats 2 (IFIT2), mRNA. |
| CD68 | 2.75 | Homo sapiens CD68 molecule (CD68), transcript variant 1, mRNA. |
| TNFSF13B | 2.66 | Homo sapiens tumor necrosis factor (ligand) superfamily, member 13b (TNFSF13B), transcript variant 1, mRNA. |
| LMO2 | 2.64 | Homo sapiens LIM domain only 2 (rhombotin-like 1) (LMO2), mRNA. |
| GCA | 2.61 | Homo sapiens grancalcin, EF-hand calcium binding protein (GCA), mRNA. |
| USP41 | 2.58 | PREDICTED: Homo sapiens ubiquitin specific peptidase 41 (USP41), mRNA. |
| GMPR | 2.56 | Homo sapiens guanosine monophosphate reductase (GMPR), mRNA. |
| ANGPTL4 | 2.56 | Homo sapiens angiopoietin-like 4 (ANGPTL4), transcript variant 1, mRNA. |
| MX1 | 2.56 | Homo sapiens myxovirus (influenza virus) resistance 1, interferon-inducible protein p78 (mouse) (MX1), mRNA. |
| HIST2H2AA3 | 2.54 | Homo sapiens histone cluster 2, H2aa3 (HIST2H2AA3), mRNA. |
| OAS2 | 2.48 | Homo sapiens 2'-5'-oligoadenylate synthetase 2, 69/71kDa (OAS2), transcript variant 2, mRNA. |
| ESM1 | 2.45 | Homo sapiens endothelial cell-specific molecule 1 (ESM1), mRNA. |
| IFI6 | 2.42 | Homo sapiens interferon, alpha-inducible protein 6 (IFI6), transcript variant 2, mRNA. |
| IRF7 | 2.38 | Homo sapiens interferon regulatory factor 7 (IRF7), transcript variant b, mRNA. |
| HPSE | 2.36 | Homo sapiens heparanase (HPSE), mRNA. |
| DTNA | 2.34 | Homo sapiens dystrobrevin, alpha (DTNA), transcript variant 7, mRNA. |
| OAS2 | 2.31 | Homo sapiens 2'-5'-oligoadenylate synthetase 2, 69/71kDa (OAS2), transcript variant 1, mRNA. |
| F3 | 2.30 | Homo sapiens coagulation factor III (thromboplastin, tissue factor) (F3), mRNA. |
| TGFB3 | 2.29 | Homo sapiens transforming growth factor, beta 3 (TGFB3), mRNA. |
| PLSCR1 | 2.28 | Homo sapiens phospholipid scramblase 1 (PLSCR1), mRNA. |
| ESM1 | 2.27 | Homo sapiens endothelial cell-specific molecule 1 (ESM1), mRNA. |
| DDX58 | 2.27 | Homo sapiens DEAD (Asp-Glu-Ala-Asp) box polypeptide 58 (DDX58), mRNA. |
| HS.553301 | 2.27 | AV737317 CB Homo sapiens cDNA clone CBCAQH03 5, mRNA sequence |
| IFI44L | 2.24 | Homo sapiens interferon-induced protein 44-like (IFI44L), mRNA. |
| HIST2H2AA3 | 2.23 | Homo sapiens histone cluster 2, H2aa3 (HIST2H2AA3), mRNA. |
| LGALS9 | 2.22 | Homo sapiens lectin, galactoside-binding, soluble, 9 (LGALS9), transcript variant 1, mRNA. |
| HIST2H2AA4 | 2.21 | Homo sapiens histone cluster 2, H2aa4 (HIST2H2AA4), mRNA. |
| IRF7 | 2.20 | Homo sapiens interferon regulatory factor 7 (IRF7), transcript variant b, mRNA. |
| CD68 | 2.18 | Homo sapiens CD68 antigen (CD68), mRNA. |
| MMP10 | 2.18 | Homo sapiens matrix metallopeptidase 10 (stromelysin 2) (MMP10), mRNA. |
| DKK1 | 2.14 | Homo sapiens dickkopf homolog 1 (Xenopus laevis) (DKK1), mRNA. |
| ISG20 | 2.14 | Homo sapiens interferon stimulated exonuclease gene 20kDa (ISG20), mRNA. |
| OAS1 | 2.11 | Homo sapiens 2',5'-oligoadenylate synthetase 1, 40/46kDa (OAS1), transcript variant 1, mRNA. |
| DUSP6 | 2.09 | Homo sapiens dual specificity phosphatase 6 (DUSP6), transcript variant 1, mRNA. |
| TACSTD2 | 2.08 | Homo sapiens tumor-associated calcium signal transducer 2 (TACSTD2), mRNA. |
| TFRC | 2.06 | Homo sapiens transferrin receptor (p90, CD71) (TFRC), mRNA. |
| SLC15A3 | 2.05 | Homo sapiens solute carrier family 15, member 3 (SLC15A3), mRNA. |
| LNPEP | 2.04 | Homo sapiens leucyl/cystinyl aminopeptidase (LNPEP), transcript variant 2, mRNA. |
| RHOB | 2.03 | Homo sapiens ras homolog gene family, member B (RHOB), mRNA. |
| IFIT3 | 2.03 | Homo sapiens interferon-induced protein with tetratricopeptide repeats 3 (IFIT3), mRNA. |
| HIST3H2A | 2.01 | Homo sapiens histone cluster 3, H2a (HIST3H2A), mRNA. |
| HSPE1 | 2.01 | Homo sapiens heat shock 10kDa protein 1 (chaperonin 10) (HSPE1), mRNA. |
| DUSP6 | 2.00 | Homo sapiens dual specificity phosphatase 6 (DUSP6), transcript variant 2, mRNA. |
| SLC3A2 | 1.99 | Homo sapiens solute carrier family 3 (activators of dibasic and neutral amino acid transport), member 2 (SLC3A2), transcript variant 6, mRNA. |
| GBP7 | 1.99 | Homo sapiens guanylate binding protein 7 (GBP7), mRNA. |
| CACNA1I | 1.99 | Homo sapiens calcium channel, voltage-dependent, T type, alpha 1I subunit (CACNA1I), transcript variant 2, mRNA. |
| PARP10 | 1.98 | Homo sapiens poly (ADP-ribose) polymerase family, member 10 (PARP10), mRNA. |
| HIST2H2AC | 1.98 | Homo sapiens histone cluster 2, H2ac (HIST2H2AC), mRNA. |
| TSPAN13 | 1.98 | Homo sapiens tetraspanin 13 (TSPAN13), mRNA. |
| PTPRU | 1.96 | Homo sapiens protein tyrosine phosphatase, receptor type, U (PTPRU), transcript variant 2, mRNA. |
| HIST2H2AB | 1.96 | Homo sapiens histone cluster 2, H2ab (HIST2H2AB), mRNA. |
| AKR1C4 | 1.95 | Homo sapiens aldo-keto reductase family 1, member C4 (chlordecone reductase; 3-alpha hydroxysteroid dehydrogenase, type I; dihydrodiol dehydrogenase 4) (AKR1C4), mRNA. |
| DOPEY1 | 1.94 | Homo sapiens dopey family member 1 (DOPEY1), mRNA. |
| FLRT3 | 1.94 | Homo sapiens fibronectin leucine rich transmembrane protein 3 (FLRT3), transcript variant 2, mRNA. |
| CD38 | 1.93 | Homo sapiens CD38 molecule (CD38), mRNA. |
| IFIH1 | 1.93 | Homo sapiens interferon induced with helicase C domain 1 (IFIH1), mRNA. |
| CTSL1 | 1.92 | Homo sapiens cathepsin L1 (CTSL1), transcript variant 1, mRNA. |
| PRIC285 | 1.91 | Homo sapiens peroxisomal proliferator-activated receptor A interacting complex 285 (PRIC285), transcript variant 2, mRNA. |
| MDK | 1.90 | Homo sapiens midkine (neurite growth-promoting factor 2) (MDK), transcript variant 1, mRNA. |
| SP110 | 1.89 | Homo sapiens SP110 nuclear body protein (SP110), transcript variant b, mRNA. |
| LUC7L | 1.89 | Homo sapiens LUC7-like (S. cerevisiae) (LUC7L), transcript variant 1, mRNA. |
| CTGF | 1.87 | Homo sapiens connective tissue growth factor (CTGF), mRNA. |
| PHACTR4 | 1.86 | Homo sapiens phosphatase and actin regulator 4 (PHACTR4), transcript variant 1, mRNA. |
| MCL1 | 1.86 | Homo sapiens myeloid cell leukemia sequence 1 (BCL2-related) (MCL1), transcript variant 1, mRNA. |
| PALLD | -1.86 | Homo sapiens palladin, cytoskeletal associated protein (PALLD), transcript variant 2, mRNA. |
| SRGN | -1.87 | Homo sapiens serglycin (SRGN), mRNA. |
| LOC100133923 | -1.87 | PREDICTED: Homo sapiens hypothetical protein LOC100133923 (LOC100133923), mRNA. |
| HLA-DMA | -1.87 | Homo sapiens major histocompatibility complex, class II, DM alpha (HLA-DMA), mRNA. |
| ALDH1A3 | -1.87 | Homo sapiens aldehyde dehydrogenase 1 family, member A3 (ALDH1A3), mRNA. |
| RHOU | -1.88 | Homo sapiens ras homolog gene family, member U (RHOU), mRNA. |
| NCALD | -1.88 | Homo sapiens neurocalcin delta (NCALD), mRNA. |
| PRR11 | -1.88 | Homo sapiens proline rich 11 (PRR11), mRNA. |
| DHCR24 | -1.88 | Homo sapiens 24-dehydrocholesterol reductase (DHCR24), mRNA. |
| HLA-DMB | -1.88 | Homo sapiens major histocompatibility complex, class II, DM beta (HLA-DMB), mRNA. |
| SLC16A2 | -1.88 | Homo sapiens solute carrier family 16, member 2 (monocarboxylic acid transporter 8) (SLC16A2), mRNA. |
| C10ORF58 | -1.89 | Homo sapiens chromosome 10 open reading frame 58 (C10orf58), transcript variant 1, mRNA. |
| ACO1 | -1.89 | Homo sapiens aconitase 1, soluble (ACO1), mRNA. |
| IGFBP7 | -1.89 | Homo sapiens insulin-like growth factor binding protein 7 (IGFBP7), mRNA. |
| SLC39A8 | -1.89 | Homo sapiens solute carrier family 39 (zinc transporter), member 8 (SLC39A8), transcript variant 1, mRNA. |
| NEK2 | -1.89 | Homo sapiens NIMA (never in mitosis gene a)-related kinase 2 (NEK2), mRNA. |
| HSD11B1 | -1.90 | Homo sapiens hydroxysteroid (11-beta) dehydrogenase 1 (HSD11B1), transcript variant 2, mRNA. |
| BRI3BP | -1.90 | PREDICTED: Homo sapiens BRI3 binding protein (BRI3BP), mRNA. |
| ALDH1A3 | -1.91 | Homo sapiens aldehyde dehydrogenase 1 family, member A3 (ALDH1A3), mRNA. |
| PLAT | -1.92 | Homo sapiens plasminogen activator, tissue (PLAT), transcript variant 1, mRNA. |
| CDCA8 | -1.92 | Homo sapiens cell division cycle associated 8 (CDCA8), mRNA. |
| SDC4 | -1.92 | Homo sapiens syndecan 4 (SDC4), mRNA. |
| RAD51AP1 | -1.92 | Homo sapiens RAD51 associated protein 1 (RAD51AP1), mRNA. |
| SLC39A8 | -1.93 | Homo sapiens solute carrier family 39 (zinc transporter), member 8 (SLC39A8), transcript variant 1, mRNA. |
| NUSAP1 | -1.93 | Homo sapiens nucleolar and spindle associated protein 1 (NUSAP1), transcript variant 2, mRNA. |
| SLC26A4 | -1.93 | Homo sapiens solute carrier family 26, member 4 (SLC26A4), mRNA. |
| UHRF1 | -1.94 | Homo sapiens ubiquitin-like with PHD and ring finger domains 1 (UHRF1), transcript variant 1, mRNA. |
| HLA-DRB1 | -1.95 | Homo sapiens major histocompatibility complex, class II, DR beta 1 (HLA-DRB1), mRNA. |
| ZWINT | -1.95 | Homo sapiens ZW10 interactor (ZWINT), transcript variant 3, mRNA. |
| CYP24A1 | -1.95 | Homo sapiens cytochrome P450, family 24, subfamily A, polypeptide 1 (CYP24A1), nuclear gene encoding mitochondrial protein, mRNA. |
| HSD11B1 | -1.95 | Homo sapiens hydroxysteroid (11-beta) dehydrogenase 1 (HSD11B1), transcript variant 2, mRNA. |
| TACC3 | -1.95 | Homo sapiens transforming, acidic coiled-coil containing protein 3 (TACC3), mRNA. |
| KIAA1199 | -1.96 | Homo sapiens KIAA1199 (KIAA1199), mRNA. |
| HLA-DRA | -1.96 | Homo sapiens major histocompatibility complex, class II, DR alpha (HLA-DRA), mRNA. |
| SCARA3 | -1.97 | Homo sapiens scavenger receptor class A, member 3 (SCARA3), transcript variant 1, mRNA. |
| HS.5724 | -1.98 | Homo sapiens mRNA; cDNA DKFZp779O0231 (from clone DKFZp779O0231) |
| STIL | -1.99 | Homo sapiens SCL/TAL1 interrupting locus (STIL), transcript variant 2, mRNA. |
| ASF1B | -1.99 | Homo sapiens ASF1 anti-silencing function 1 homolog B (S. cerevisiae) (ASF1B), mRNA. |
| FOXM1 | -2.00 | Homo sapiens forkhead box M1 (FOXM1), transcript variant 2, mRNA. |
| LPP | -2.00 | Homo sapiens LIM domain containing preferred translocation partner in lipoma (LPP), mRNA. |
| CENPK | -2.01 | Homo sapiens centromere protein K (CENPK), mRNA. |
| MNS1 | -2.01 | Homo sapiens meiosis-specific nuclear structural 1 (MNS1), mRNA. |
| ZWINT | -2.01 | Homo sapiens ZW10 interactor (ZWINT), transcript variant 3, mRNA. |
| NCCRP1 | -2.02 | Homo sapiens non-specific cytotoxic cell receptor protein 1 homolog (zebrafish) (NCCRP1), mRNA. |
| MMP9 | -2.03 | Homo sapiens matrix metallopeptidase 9 (gelatinase B, 92kDa gelatinase, 92kDa type IV collagenase) (MMP9), mRNA. |
| DIAPH3 | -2.05 | Homo sapiens diaphanous homolog 3 (Drosophila) (DIAPH3), transcript variant 1, mRNA. |
| DTL | -2.05 | Homo sapiens denticleless homolog (Drosophila) (DTL), mRNA. |
| LIPG | -2.05 | Homo sapiens lipase, endothelial (LIPG), mRNA. |
| CDC2 | -2.05 | Homo sapiens cell division cycle 2, G1 to S and G2 to M (CDC2), transcript variant 1, mRNA. |
| GINS2 | -2.06 | Homo sapiens GINS complex subunit 2 (Psf2 homolog) (GINS2), mRNA. |
| HS.531457 | -2.06 | Homo sapiens cDNA FLJ37595 fis, clone BRCOC2007864 |
| FAM64A | -2.06 | Homo sapiens family with sequence similarity 64, member A (FAM64A), mRNA. |
| MAD2L1 | -2.07 | Homo sapiens MAD2 mitotic arrest deficient-like 1 (yeast) (MAD2L1), mRNA. |
| TPX2 | -2.07 | Homo sapiens TPX2, microtubule-associated, homolog (Xenopus laevis) (TPX2), mRNA. |
| KIFC1 | -2.08 | Homo sapiens kinesin family member C1 (KIFC1), mRNA. |
| HEG1 | -2.08 | Homo sapiens HEG homolog 1 (zebrafish) (HEG1), mRNA. |
| HJURP | -2.08 | Homo sapiens Holliday junction recognition protein (HJURP), mRNA. |
| MELK | -2.10 | Homo sapiens maternal embryonic leucine zipper kinase (MELK), mRNA. |
| CCNB2 | -2.10 | Homo sapiens cyclin B2 (CCNB2), mRNA. |
| CDKN3 | -2.10 | Homo sapiens cyclin-dependent kinase inhibitor 3 (CDK2-associated dual specificity phosphatase) (CDKN3), mRNA. |
| HSD11B1 | -2.11 | Homo sapiens hydroxysteroid (11-beta) dehydrogenase 1 (HSD11B1), transcript variant 1, mRNA. |
| SYNC1 | -2.11 | Homo sapiens syncoilin, intermediate filament 1 (SYNC1), mRNA. |
| BUB1 | -2.11 | Homo sapiens BUB1 budding uninhibited by benzimidazoles 1 homolog (yeast) (BUB1), mRNA. |
| DIAPH3 | -2.12 | Homo sapiens diaphanous homolog 3 (Drosophila) (DIAPH3), transcript variant 1, mRNA. |
| HLA-DPA1 | -2.13 | Homo sapiens major histocompatibility complex, class II, DP alpha 1 (HLA-DPA1), mRNA. |
| UBD | -2.13 | Homo sapiens ubiquitin D (UBD), mRNA. |
| SH3PXD2A | -2.13 | Homo sapiens SH3 and PX domains 2A (SH3PXD2A), mRNA. |
| CCNA2 | -2.13 | Homo sapiens cyclin A2 (CCNA2), mRNA. |
| SAA1 | -2.15 | Homo sapiens serum amyloid A1 (SAA1), transcript variant 2, mRNA. |
| CXCL9 | -2.17 | Homo sapiens chemokine (C-X-C motif) ligand 9 (CXCL9), mRNA. |
| PSMC3IP | -2.18 | Homo sapiens PSMC3 interacting protein (PSMC3IP), transcript variant 2, mRNA. |
| TRIP13 | -2.19 | Homo sapiens thyroid hormone receptor interactor 13 (TRIP13), mRNA. |
| CDCA5 | -2.19 | Homo sapiens cell division cycle associated 5 (CDCA5), mRNA. |
| FABP3 | -2.19 | Homo sapiens fatty acid binding protein 3, muscle and heart (mammary-derived growth inhibitor) (FABP3), mRNA. |
| HADH | -2.20 | Homo sapiens hydroxyacyl-Coenzyme A dehydrogenase (HADH), nuclear gene encoding mitochondrial protein, mRNA. |
| CYP1B1 | -2.21 | Homo sapiens cytochrome P450, family 1, subfamily B, polypeptide 1 (CYP1B1), mRNA. |
| CDC45L | -2.21 | Homo sapiens CDC45 cell division cycle 45-like (S. cerevisiae) (CDC45L), mRNA. |
| KIF11 | -2.23 | Homo sapiens kinesin family member 11 (KIF11), mRNA. |
| NDC80 | -2.24 | Homo sapiens NDC80 homolog, kinetochore complex component (S. cerevisiae) (NDC80), mRNA. |
| HAS3 | -2.25 | Homo sapiens hyaluronan synthase 3 (HAS3), transcript variant 1, mRNA. |
| HMMR | -2.26 | Homo sapiens hyaluronan-mediated motility receptor (RHAMM) (HMMR), transcript variant 2, mRNA. |
| SCARA3 | -2.26 | Homo sapiens scavenger receptor class A, member 3 (SCARA3), transcript variant 2, mRNA. |
| LOC730415 | -2.29 | PREDICTED: Homo sapiens hypothetical LOC730415, transcript variant 2 (LOC730415), mRNA. |
| APOBEC3B | -2.30 | Homo sapiens apolipoprotein B mRNA editing enzyme, catalytic polypeptide-like 3B (APOBEC3B), mRNA. |
| KIF23 | -2.31 | Homo sapiens kinesin family member 23 (KIF23), transcript variant 2, mRNA. |
| AURKB | -2.32 | Homo sapiens aurora kinase B (AURKB), mRNA. |
| HLA-DRB4 | -2.36 | Homo sapiens major histocompatibility complex, class II, DR beta 4 (HLA-DRB4), mRNA. |
| ASPM | -2.38 | Homo sapiens asp (abnormal spindle) homolog, microcephaly associated (Drosophila) (ASPM), mRNA. |
| BIRC5 | -2.43 | Homo sapiens baculoviral IAP repeat-containing 5 (BIRC5), transcript variant 1, mRNA. |
| DLGAP5 | -2.46 | Homo sapiens discs, large (Drosophila) homolog-associated protein 5 (DLGAP5), mRNA. |
| CDCA3 | -2.47 | Homo sapiens cell division cycle associated 3 (CDCA3), mRNA. |
| KIF4A | -2.49 | Homo sapiens kinesin family member 4A (KIF4A), mRNA. |
| CEP55 | -2.51 | Homo sapiens centrosomal protein 55kDa (CEP55), mRNA. |
| CDC20 | -2.52 | Homo sapiens cell division cycle 20 homolog (S. cerevisiae) (CDC20), mRNA. |
| RASL11B | -2.52 | Homo sapiens RAS-like, family 11, member B (RASL11B), mRNA. |
| TYMS | -2.53 | Homo sapiens thymidylate synthetase (TYMS), mRNA. |
| PBK | -2.54 | Homo sapiens PDZ binding kinase (PBK), mRNA. |
| METTL7A | -2.55 | Homo sapiens methyltransferase like 7A (METTL7A), mRNA. |
| KIAA0101 | -2.59 | Homo sapiens KIAA0101 (KIAA0101), transcript variant 1, mRNA. |
| NUSAP1 | -2.61 | Homo sapiens nucleolar and spindle associated protein 1 (NUSAP1), transcript variant 2, mRNA. |
| PRC1 | -2.65 | Homo sapiens protein regulator of cytokinesis 1 (PRC1), transcript variant 2, mRNA. |
| TK1 | -2.68 | Homo sapiens thymidine kinase 1, soluble (TK1), mRNA. |
| ANLN | -2.69 | Homo sapiens anillin, actin binding protein (ANLN), mRNA. |
| RRM2 | -2.77 | Homo sapiens ribonucleotide reductase M2 polypeptide (RRM2), mRNA. |
| KIF11 | -2.79 | Homo sapiens kinesin family member 11 (KIF11), mRNA. |
| NCAPG | -2.84 | Homo sapiens non-SMC condensin I complex, subunit G (NCAPG), mRNA. |
| HMMR | -2.86 | Homo sapiens hyaluronan-mediated motility receptor (RHAMM) (HMMR), transcript variant 1, mRNA. |
| CDC2 | -2.98 | Homo sapiens cell division cycle 2, G1 to S and G2 to M (CDC2), transcript variant 1, mRNA. |
| UBE2C | -3.02 | Homo sapiens ubiquitin-conjugating enzyme E2C (UBE2C), transcript variant 6, mRNA. |
| TOP2A | -3.14 | Homo sapiens topoisomerase (DNA) II alpha 170kDa (TOP2A), mRNA. |
| CD74 | -3.18 | Homo sapiens CD74 molecule, major histocompatibility complex, class II invariant chain (CD74), transcript variant 2, mRNA. |
| UBE2C | -3.18 | Homo sapiens ubiquitin-conjugating enzyme E2C (UBE2C), transcript variant 3, mRNA. |
| MYH11 | -3.27 | Homo sapiens myosin, heavy chain 11, smooth muscle (MYH11), transcript variant SM1A, mRNA. |
| CD74 | -3.48 | Homo sapiens CD74 molecule, major histocompatibility complex, class II invariant chain (CD74), transcript variant 1, mRNA. |
| HIST1H4C | -3.58 | Homo sapiens histone cluster 1, H4c (HIST1H4C), mRNA. |

Table S3. Top 11 genes regulated by OEO in the canonical mismatch repair in eukaryotes pathway. Fold change over vehicle was shown in log_2_ ratio form.


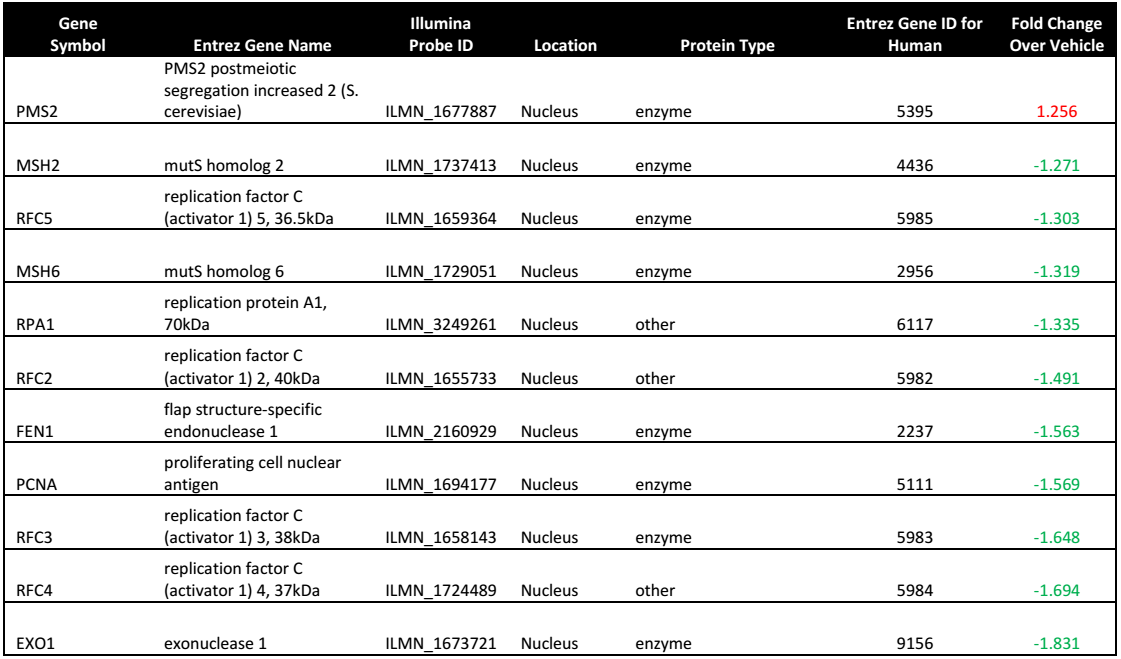


Table S4. Top 20 genes regulated by OEO in the canonical pathway of the role of BRCA1 in DNA damage response. Fold change over vehicle was shown in log_2_ ratio form.
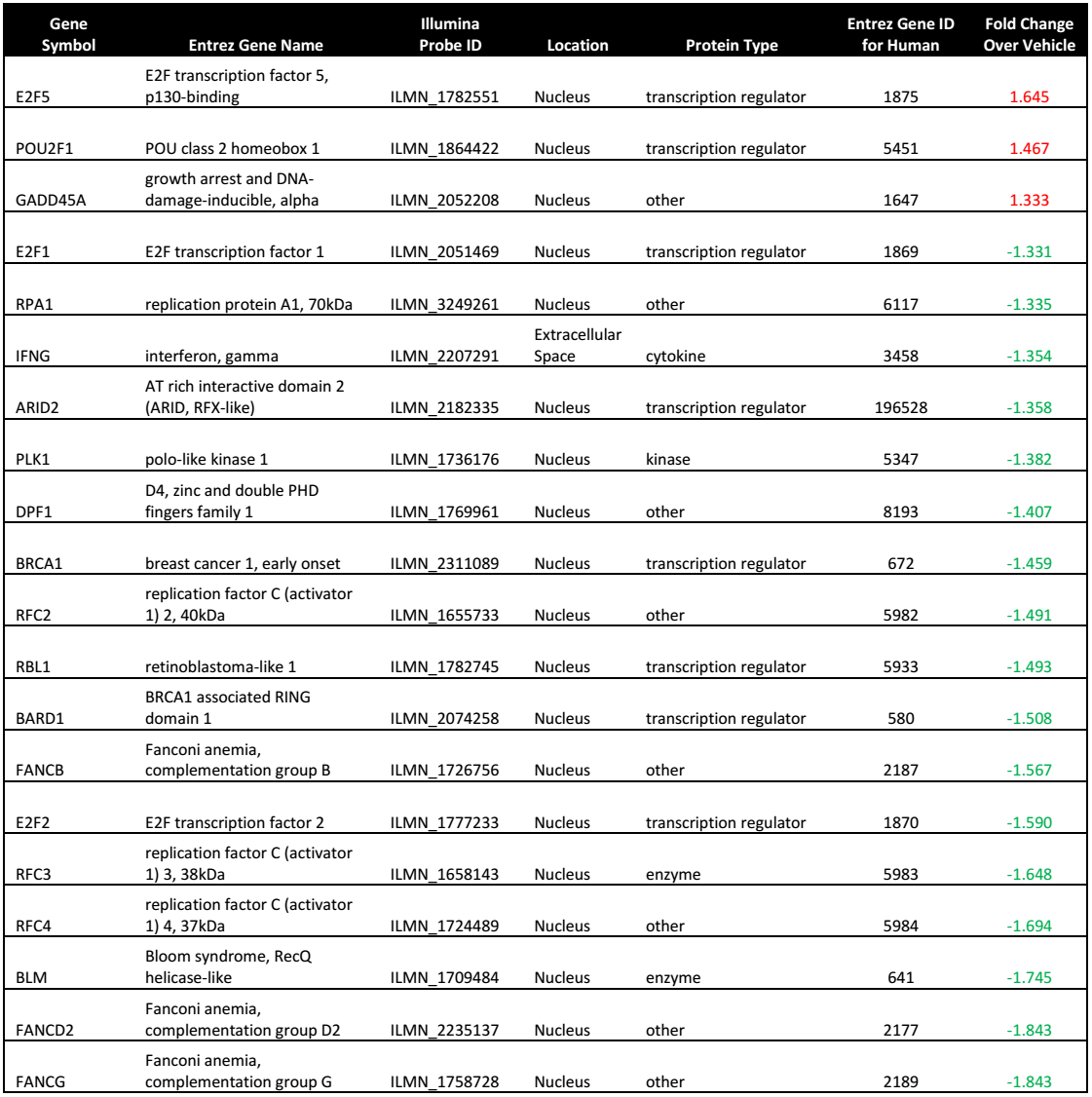


Table S5. Top 15 genes regulated by OEO in the canonical cell cycle control of chromosomal replication pathway. Fold change over vehicle was shown in log_2_ ratio form.


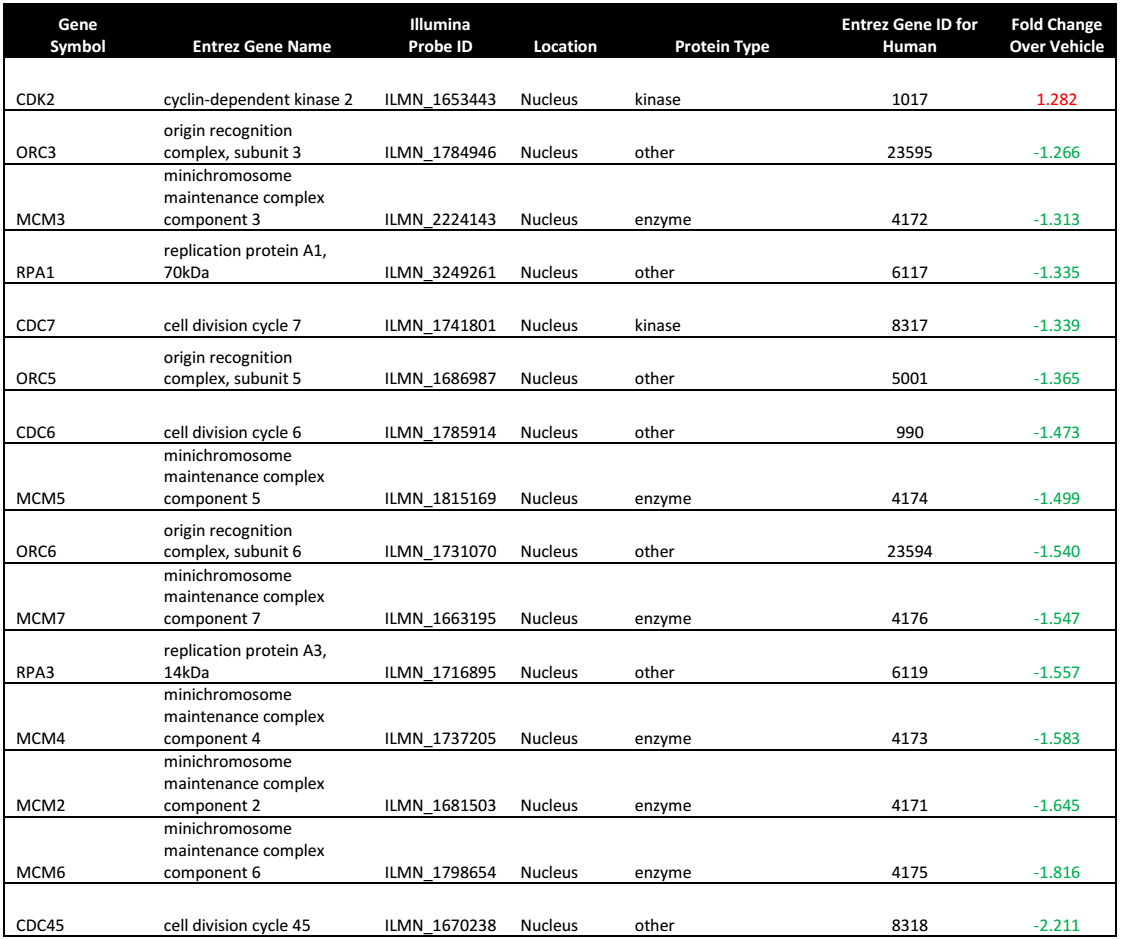


Table S6. Top 20 genes regulated by OEO in the canonical role of CHK proteins in cell cycle checkpoint control pathway. Fold change over vehicle was shown in log_2_ ratio form.
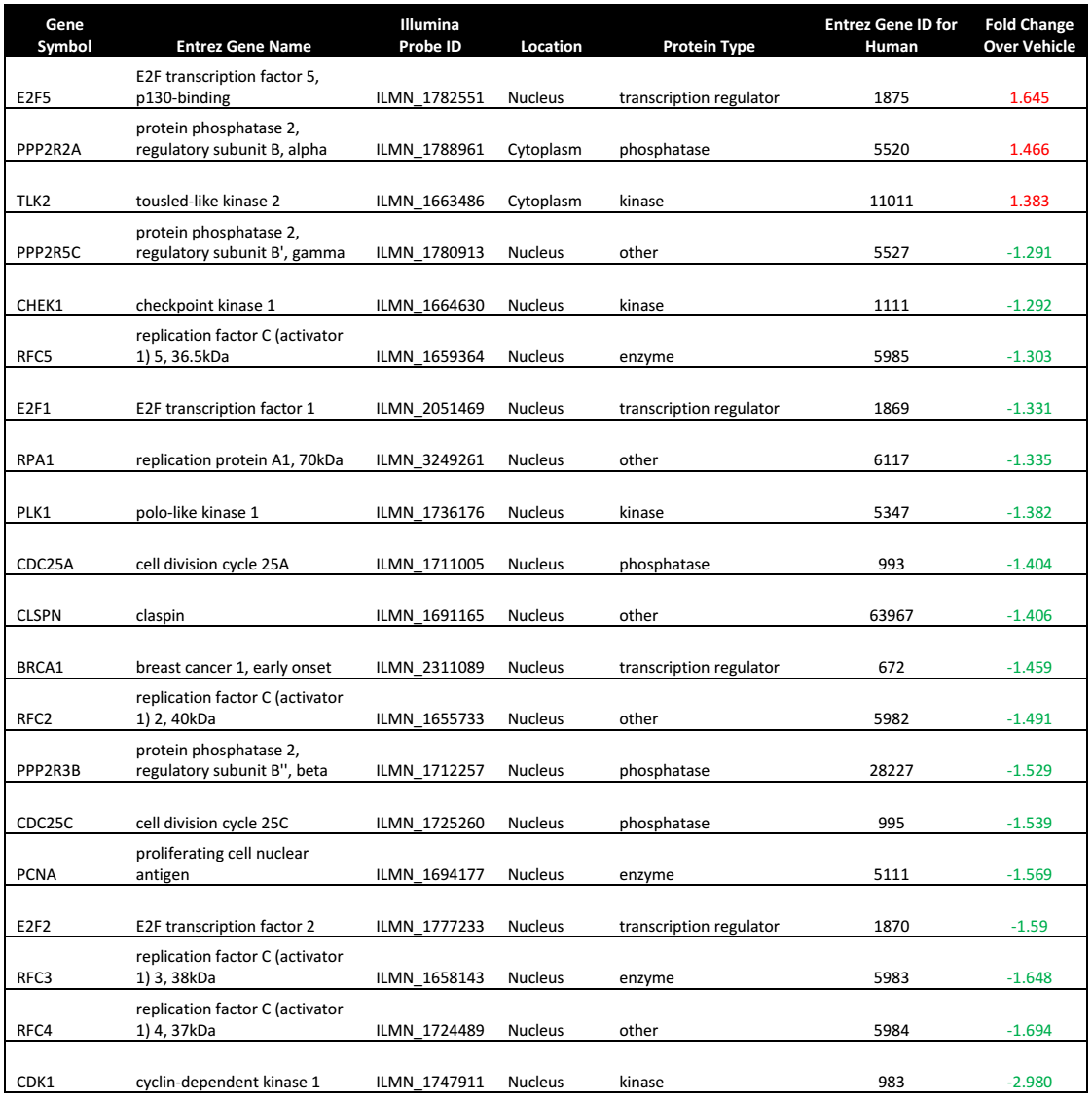

Supplement: Supplementary file 1 [file mmc1.docx]
